# Akt regulates the expression of MafK, synaptotagmin I, and syntenin-1, which play roles in neuronal function

**DOI:** 10.1186/1423-0127-17-18

**Published:** 2010-03-17

**Authors:** Young-Tae Ro, Bo-Kwang Jang, Chan Young Shin, Eui U Park, Chul Geun Kim, Sung-Il Yang

**Affiliations:** 1 Department of Biochemistry, School of Medicine, Konkuk University, Seoul, Republic of Korea; 2 Department of Pharmacology, School of Medicine, Konkuk University, Seoul, Republic of Korea; 3 Department of Forensic Medical Science, School of Medicine, Konkuk University, Seoul, Republic of Korea; 4 Research Institute of Medical Science, Konkuk University, Seoul, Republic of Korea; 5 Department of Life Science, College of Natural Sciences, Hanyang University, Seoul, Republic of Korea

## Abstract

**Background:**

Akt regulates various cellular processes, including cell growth, survival, and metabolism. Recently, Akt's role in neurite outgrowth has also emerged. We thus aimed to identify neuronal function-related genes that are regulated by Akt.

**Methods:**

We performed suppression subtractive hybridization on two previously established PC12 sublines, one of which overexpresses the wild-type (WT) form and the other, the dominant-negative (DN) form of Akt. These sublines respond differently to NGF's neuronal differentiation effect.

**Results:**

A variety of genes was identified and could be classified into several functional groups, one of which was developmental processes. Two genes involved in neuronal differentiation and function were found in this group. v-Maf musculoaponeurotic fibrosarcoma oncogene homolog K (MafK) induces the neuronal differentiation of PC12 cells and immature telencephalon neurons, and synaptotagmin I (SytI) is essential for neurotransmitter release. Another gene, *syntenin-1 *(*Syn-1*) was also recognized in the same functional group into which *MafK *and *SytI *were classified. Syn-1 has been reported to promote the formation of membrane varicosities in neurons. Quantitative reverse transcription polymerase chain reaction analyses show that the transcript levels of these three genes were lower in PC12 (WT-Akt) cells than in parental PC12 and PC12 (DN-Akt) cells. Furthermore, treatment of PC12 (WT-Akt) cells with an Akt inhibitor resulted in the increase of the expression of these genes and the improvement of neurite outgrowth. These results indicate that dominant-negative or pharmacological inhibition of Akt increases the expression of *MafK*, *SytI*, and *Syn-1 *genes. Using lentiviral shRNA to knock down endogenous Syn-1 expression, we demonstrated that Syn-1 promotes an increase in the numbers of neurites and branches.

**Conclusions:**

Taken together, these results indicate that Akt negatively regulates the expression of *MafK*, *SytI*, and *Syn-1 *genes that all participate in regulating neuronal integrity in some way or another.

## Background

Akt (also termed "protein kinase B') mediates a variety of biological responses to insulin, cytokines, and numerous growth factors. As such, Akt has been well recognized as an important regulator for multiple biological processes, including metabolism, cell size, apoptosis, and cell cycle progression [1]. Recently, the importance of Akt in neuronal functions beyond neuronal protection against apoptotic insults has emerged. Akt was reported to inhibit the neuronal differentiation of hippocampal neural progenitor cells [2] and of PC12 cells [3-5]. Similarly, Akt activity was found to be sustainedly augmented when neurite outgrowth of PC12 cells was inhibited by CSK overexpression [6].

These actions of Akt are evoked by phosphorylating its substrates and thus regulating the activity of proteins and the expression of genes. A number of Akt substrates and Akt-regulated genes have been identified, but these are mostly involved in metabolism, cell size, apoptosis, and cell cycle progression. These include Gsk3, BAD, p21^Cip1/WAF1^, p27^Kip1^, and certain transcription factors and transcription factor regulators such as cAMP-response element binding protein (CREB), the FOXO family of Forkhead transcription factors, IκB kinase, and Mdm2 [7-16]. Through these transcription factors and regulators, Akt regulates the transcription of genes that possess anti-apoptotic, pro-survival or pro-apoptotic functions, such as *Bcl-2*, Bcl-_XL_, A1, and FasL [15,17,18].

Unlike these Akt-regulated genes in apoptosis and survival, however, hardly any genes implicated in neuronal differentiation process have been revealed to be regulated by Akt. Therefore, we sought to find Akt-regulated differentiation genes in rat PC12 pheochromocytoma cells, which are often used as a model of neuronal differentiation. We performed suppression subtractive hybridization (SSH) on two previously established subclonal PC12 cell lines that ectopically express a wild-type (WT) or dominant-negative (DN) form of Akt1 [3]. PC12 (WT-Akt) cells barely differentiate in response to NGF, whereas PC12 (DN-Akt) cells extend their neurites quite well. Approximately seventy genes including *v-maf musculoaponeurotic fibrosarcoma oncogene homolog K *(*MafK*), *synaptotagmin I *(*SytI*), and *syntenin-1 *(*Syn-1*) were recognized as genes expressed at a higher level in cells that express have PC12 (DN-Akt) cells. We demonstrated here that knockdown of Syn-1 decreases the number of neurites per neuritogenic cell and the percentage of branch-bearing neurites, implicating a positive role for Syn-1 in neurite outgrowth. Likewise, MafK and SytI are known to play a role in neuronal differentiation and neurotransmitter release, respectively. By quantitative reverse transcription polymerase chain reaction (RT-PCR) analysis, we confirmed that PC12 (WT-Akt) cells had a lower level of expression of these three genes than PC12 (WT-Akt) cells, and also demonstrated that pharmacological inhibition of Akt causes an increase in the expression of these genes in PC12 (WT-Akt) cells. Therefore, it is alleged that Akt downregulates the expression of *MafK*, *SytI*, and *Syn-1 *genes, all of which are positively related to neuronal function.

## Methods

### Materials

A potent and specific inhibitor of Akt (AKTi-1/2; naphthyridinone) and the Gsk3β inhibitor TWS119 were purchased from Calbiochem (CA, USA). Actinomycin D was from Sigma (MO, USA). Nerve growth factor (NGF) 2.5 S was obtained from Roche (NJ, USA) and the iQ™ SYBR^® ^Green Supermix came from Bio-Rad (CA, USA).

### Cell culture

The PC12-derived subclones, PC12 (WT-Akt) and PC12 (DN-Akt), were isolated as previously described [3]. Cells were grown in DMEM containing 10% horse serum and 5% fetal bovine serum in a 5% CO_2 _atmosphere. For maintenance of the subclones, 200 μg/ml of G418 was included in the culture media. Where indicated, 100 ng/ml of NGF was added to the cultures in DMEM containing 1% horse serum and 0.5% fetal calf serum.

### SSH

Cells were treated with 100 ng/ml of NGF for 2 hrs. Total RNA was prepared with the RNeasy kit (Qiagen, CA, USA) and cDNA was synthesized using the SMART™ PCR cDNA Synthesis Kit (BD Biosciences, CA, USA). SSH was carried out with the PCR-Select™ cDNA subtraction kit (BD Biosciences), as described by the manufacturer. cDNAs synthesized from the total RNA of PC12 (WT-Akt) and PC12 (DN-Akt) cells were used as the drivers and testers, respectively. Plasmids encoding subtracted cDNA were subjected to cycle sequencing using the ABI PRISM™ 310 genetic analyzer (Applied Biosystems, CA, USA). The sequences obtained were used for sequence alignment with the National Center for Biotechnology Information, GenBank, using a basic blast search.

### Semi-quantitative and quantitative RT-PCR

cDNA was synthesized from RNA using the Omniscript RT kit (Qiagen) according to the manufacturer's protocol. The cDNA mixture was amplified using PCR with specific primers (Table 1), and the resulting products were separated on 2% agarose gel. Quantitative PCR was run using SYBR green PCR mastermix and the LightCycler Real-time PCR Detection System (Roche). The number of amplification steps required to reach the threshold cycle number (Ct) was computed using LightCycler software 4.0 (Roche). To normalize input cDNA, *Gapdh *was run separately in all experiments under the same conditions. The expression levels of each gene were normalized against *Gapdh *using the comparative Ct method.

**Table 1:** Primers used for semi-quantitative RT-PCR

Gene	Forward primer	Reverse primer
Ect2	5'-GAGAGCTGGCTGAAGATGCT-3'	5'-TCCTCTGAGCTATGGGATG-3'
Cadm1	5'-TCTGGGCCAAACCTGTTCATC AAT-3'	5'-CTGTGTCTGCGTCTGCTGCGTCAT-3'
Rgs4	5'-GTTGCTCCCGCTGCCTTTCTCT-3'	5'-CATGTTCCGGCTTGTCTCCTCTC-3'
Cldnd1	5'-AACTTGGGATTGTGGAGACG-3'	5'-CAGAGGTAGGTCCGAAGCAG-3'
Kntc2	5'-CCAGTTAGCTGTGCAGACCA-3'	5'-CTTCAGACGTGCATTCCTCA-3'
Socs5	5'-GTGGACATGAACGCCAACAG-3'	5'-AATCTCTGCGGCACAGTTTTG-3'
Pbrm1-predicted	5'-TGCCCTTGTCCTCCATAAAG-3'	5'-AAAGTTCGGATCCACAGCAG-3'
Apool	5'-TGAAAAATGGGGTCATGGAT-3'	5'-CTAATGTGGCCAGTCCCAGT-3'
MafK	5'-CCCAAGCCCAACAAGACATT-3'	5'-GCTGCTTCAGCCGAGTTACC-3'
SytI	5'-CCGGACTGACTGATGGAGAAG-3'	5'-ATCGGATGTACCCCCCATGT-3'
Syn-1	5'-CTCAAACTGCCTCTTCTGCTAATC-3'	5'-ACTGTCCGTTCAAAGGGTCTATC-3'
Gapdh	5'-GGTTACCAGGGCTGCCTTCT-3'	5'-ATGGGTTTCCCGTTGATGAC-3'

### Inhibitor treatment

Cells were pretreated with the Akt inhibitor AKTi-1/2 (0.1 - 10 μM) or the Gsk3β inhibitor TWS119 (10 μM) for 3 hrs in DMEM and 1% horse serum and 0.5% fetal calf serum, prior to treatment with 100 ng/ml of NGF for either 10 min or 2 hr. For the transcriptional inhibitor actinomycin D, cells were treated with 100 ng/ml of NGF for 2 hr, followed by 10 μM actinomycin D for 2 hr in the absence of NGF.

### Lentivirus vector and infection

The lentiviral shRNA vector was constructed in plasmid pLV-TH (Dr. Didier Trono, University of Geneva, Geneva, Switzerland). Target sequences for knockdown of Syn-1 were: shSyn-1#1 (GGATTAGGAGAGCAGAGAT), shSyn-1#2 (GTGAGATCAACGGACAGAA). A nonsilencing control shRNA (shControl) was GCGTGTACATGCGTGTA. Viral stock was generated by transfecting each lentiviral vector into 293T cells using a standard calcium phosphate precipitation technique. The appropriate lentiviral vector plasmid (10 μg) and the packaging vector plasmids (3.5 μg of pMD.G and 6.5 μg of pΔ8.2) were co-transfected. Viral supernatants were harvested at 48 and 72 hr after transfection and filtered through a 0.45 μm pore size filter. Viral supernatants were concentrated by centrifugation for 90 min at 50,000 × *g *and the viral particles were then resuspended in DMEM containing 10% FBS. Transduction of PC12 cells was performed at an MOI of 10 in the presence of 4 μg/ml polybrene (Sigma), and three days after infection, knockdown of Syn-1 transcript and protein was confirmed by quantitative PCR and western blot analysis.

### Evaluation of neurite outgrowth

PC12 (WT-Akt) cells were incubated with DMEM containing 1% horse serum and 0.5% fetal calf serum overnight and treated with 100 ng/ml of NGF and AKTi-1/2 (0, 0.1, 1 μM). After four days, cells with neurites of at least two cell body diameters were counted. A minimum of 50 to 100 cells per field was examined in six to nine fields. For PC12 (DN-Akt) cells, cells were infected with lentivirus targeting Syn-1 (shSyn-1#2) or shControl at an MOI of 10. Three days after infection, 10^4 ^cells were plated into a 12-well plate. Cells were incubated with DMEM containing 1% horse serum and 0.5% fetal calf serum overnight and treated with NGF for five days. GFP-expressing neuritogenic cells were examined using phase-contrast fluorescent microscopy. Ten to fifteen microscopic fields of cells were randomly chosen, and cells that had neurites exceeding twice the length of the cell body were regarded as neuritogenic cells.

### Western blot analysis

Cells were lysed as described previously [3,19], and proteins were separated with SDS-10% PAGE and transferred onto PVDF membranes. Membranes were incubated with antibodies for Akt (Cell Signaling Technology, MA, USA), SytI (BD Biosciences), MafK/NF-E2 p18 (Santa Cruz Biotechnology, CA, USA), Syn-1 (Millipore, CA, USA), β-catenin (Santa Cruz Biotechnology), and β-Actin (Millipore). Proteins were visualized using an Enhanced Chemiluminescence kit (Intron, Seoul, Korea).

### Statistical analysis

All data are presented as means ± SD. We performed a Student t- test with a two-tailed distribution.

## Results

### Identification of genes that had lower transcript levels in PC12 (WT-Akt) cells than in PC12 (DN-Akt) cells

**Figure 1:**
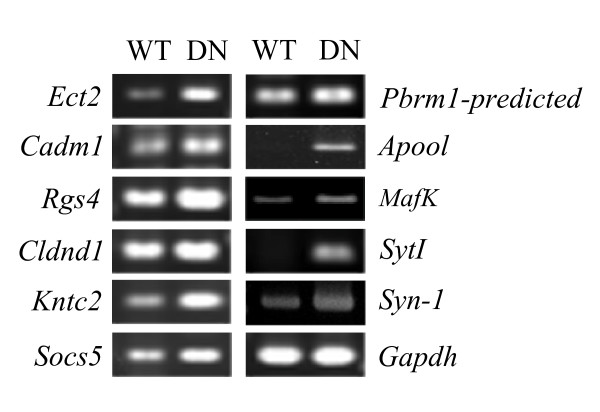
**Verification of genes identified from SSH by semi-quantitative RT-PCR**. WT, PC12 (WT-Akt) cells; DN, PC12 (DN-Akt) cells.

We previously established stable PC12 cell lines that constitutively overexpress WT-Akt1 or DN-Akt1 [3]. These sublines are designated as PC12 (WT-Akt) or PC12 (DN-Akt). These two PC12 sublines have different neurite outgrowth phenotypes from each other. PC12 (WT-Akt) cells barely differentiate in response to NGF, whereas PC12 (DN-Akt) cells have an accelerated rate of neurite outgrowth [3]. These two PC12 sublines were chosen in the present study for this reason. We attempted to first find the genes that are expressed differentially between the two subline cells and then identify those that are implicated in neuronal differentiation among Akt-regulated genes. To this end, SSH was performed using PC (DN-Akt) cells as a tester and PC (WT-Akt) cells as a driver. Approximately 70 colonies were isolated and amplified for sequencing. Semi-quantitative RT-PCR analysis validated the elevation of the transcript levels of the majority of these genes in the PC12 (DN-Akt) cells, as compared with the transcript levels in PC12 (WT-Akt) cells (Fig. 1). These genes could be categorized into five functional groups using the Database for Annotation, Visualization and Integrated Discovery (DAVID) [20]. These genes encode proteins that are involved in (1) signaling cascades and cell communication, (2) the organization and localization of cellular compartments and components, (3) gene expression, (4) protein metabolism, and (5) developmental processes (Table 2).

**Table 2:** A list of representative genes that are upregulated in PC12 (DN-Akt) cells compared to PC12 (WT-Akt) cells

Functional classification	Functional classification
*Signaling cascades, Cell communication* Epithelial cell transforming sequence 2 oncogene (Ect2) Regulator of G protein signaling 4 (Rgs4) Mss4 Syntenin 1 (Syn-1) Kinetochore associated 2_predicted (Kntc2) Synaptotagmin I (SytI) Connexin 45 (Cx45) *Organization and localization of cellular compartments and components* SytI Calnexin (Canx) Cell adhesion molecule 1 (Cadm1) Mss4 Syn-1 Kntc2 *Not clustered* Caludin domain containing 1 (Cldnd1) Similar to golli-interacting protein DEAD box polypeptide 42 Apolipoprotein O-like (Apool) Suppressor of cytokine signaling 5 (Socs5) Sp3 transcription factor	*Gene expression, Nucleic acid metabolism* DEAH box polypeptide 15 Polybromo 1_predicted (Pbrm1_predicted) v-maf musculoaponeurotic fibrosarcoma oncogene homolog K (MafK) RNA binding motif protein 3 Phosphatidylinositol glycan anchor biosynthesis, class O *Protein metabolism* SytI Canx SFRS protein kinase 2 Ect2 Methionine adenosyltransferase II, alpha (Mat2a) *Development of anatomical structure and nervous system, Developmental process, Secretion* Canx Cadm1 MafK Syn-1 SytI

Three genes, *MafK*, *SytI*, and *Syn-1*, in the fifth group of clustering drew our interest because these genes are known or presumed to be associated with neuronal function. MafK is one of the small Maf family proteins. It is expressed in neural tissues of both late embryonal stage and postnatal period, in the embryonic mesoderm, and in mesenchymal and hematopoietic cells [21]. MafK certainly plays a role in differentiation of these specific types of cells; it participates in NGF-promoted neuritogenesis of PC12 cells and immature telencephalon neurons [22], and is required for erythroid differentiation [23]. SytI is abundant in the synaptic vesicles of neurons, where it acts as a calcium sensor and plays a critical role in neurotransmitter release [24,25]. Its expression was shown to be induced in neural crest cultures by BMP4, a factor that evokes noradrenergic differentiation [26,27]. Syn-1 is involved mainly in the regulation of plasma membrane dynamics in neuronal as well as non-neuronal cells [28,29]. Ectopic expression of Syn-1 in neurons was shown to increase the number of neuritic varicosities [30].

**Figure 2:**
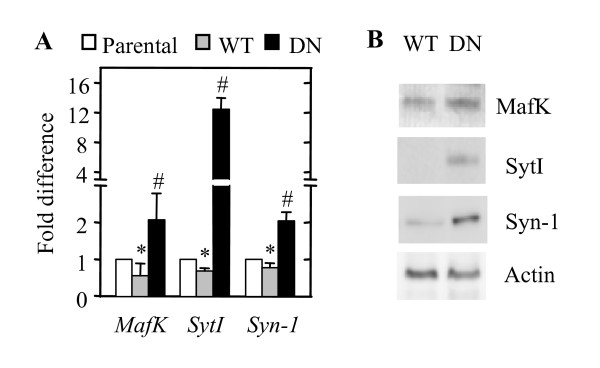
**Analyses of *MafK*, *SytI*, and *Syn-1 *mRNA levels in parental, WT-Akt-expressing, and DN-Akt-expressing PC12 cells**. (A) Quantitative RT-PCR. mRNA level of the gene in each group of cells was normalized to the level of GAPDH mRNA and the transcript level of each gene in PC12 (WT-Akt) and PC12 (DN-Akt) cells is presented as a fold change from that of the PC12 (parental) cells. Parental, PC12 (parental) cells. *P < 0.05 (parental vs. WT). *P < 0.05 (parental vs. DN). n = 3-4, mean + SD. (B) Western blot analysis of SytI and Syn-1 protein levels in PC12 (WT-Akt) and PC12 (DN-Akt) cells.

We performed quantitative RT-PCR to quantitate the differences between transcript levels in three cell populations: PC12 (WT-Akt), PC12 (DN-Akt), and PC12 (parental). The transcript level of each gene in PC12 (WT-Akt) and PC12 (DN-Akt) cells was presented as a fold change from that of the PC12 (parental) cells. As shown in Fig. 2A, all of the three genes exhibited increased transcript levels in both PC12 (DN-Akt) and PC12 (parental) cells compared to PC12 (WT-Akt) cells. The transcript levels of these genes seem inversely correlated with the Akt activity levels, being highest in PC12 (DN-Akt) and lowest in PC12 (WT-Akt). The SytI transcript was remarkably elevated, with an ~18-fold increase in PC12 (DN-Akt) cells, compared to PC12 (WT-Akt) cells. The fold differences of other gene transcripts were 2.7-4.3. To check whether detected changes in the transcript levels are followed by changes in protein levels, western blot analysis was performed (Fig. 2B). In agreement with the transcript levels, MafK, SytI, and Syn-1 protein levels were also higher in PC (DN-Akt) cells than in PC (WT-Akt) cells.

### Effect of an Akt-specific inhibitor on expression of MafK, SytI, and Syn-1 genes in PC12 (WT-Akt) cells

**Figure 3:**
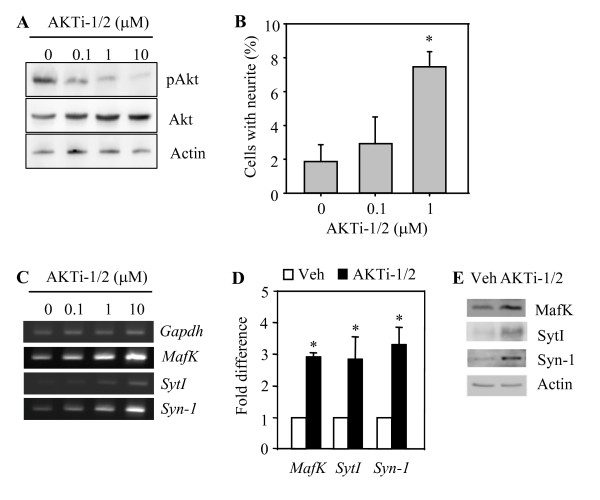
**Effect of the Akt inhibitor AKTi-1/2 on mRNA levels for the *MafK*, *SytI*, and *Syn-1 *genes**. (A) Western blot analysis of pAkt(Ser-473) protein level in PC12 (WT-Akt) cells treated with various concentrations of AKTi-1/2 prior to 10 min treatment with NGF. (B) The percentage of neuritogenic PC12 (WT-Akt) cells treated with or without AKTi-1/2. Averages and standard deviations are derived from more than six fields of view. *P < 0.001 (n = 6-9, mean + SD). (C) Semi-quantitative RT-PCR. PC12 (WT-Akt) cells were treated with various concentrations of AKTi-1/2. (D) Quantitative RT-PCR. PC12 (WT-Akt) cells were treated with 10 μM of AKTi-1/2. The mRNA level is presented as a fold difference over that of vehicle-treated cells. *P < 0.05 (n = 3-4, mean + SD). (E) Western blot analysis of SytI and Syn-1 protein levels in PC12 (WT-Akt) cells treated with vehicle (Veh) or 10 μM AKTi-1/2.

As stated above, our experiments were based on two Akt-manipulated PC12 sublines that turned out to have quite different neurite outgrowth phenotypes from each other; cells overexpressing Akt do not differentiate well but the other cells that express the dominant-negative form of Akt make neurites quite well. However, the specificity of dominant-negative Akt alleles has been contentious [31,32], and the parallel use of chemical inhibitors has been perceived as a necessary experimental approach. Therefore, we aimed to determine whether an Akt inhibitor would produce a phenotype similar to that seen in cells expressing dominant-negative Akt. We used the specific inhibitor AKTi-1/2 [33]. We treated PC12 (WT-Akt) cells with 0.1, 1, or 10 μM of AKTi-1/2. As shown in Fig. 3A, the activity of Akt (phosphorylated level of Akt on Ser-473) was decreased by AKTi-1/2 in a concentration-dependent manner, indicating that this agent works effectively as an Akt inhibitor in our cell system. We next examined the effect of this Akt inhibitor on neurite outgrowth of PC12 (WT-Akt) cells. Consistent with a previous report [3], PC12 (WT-Akt) cells did not differentiate in response to NGF. However, addition of AKTi-1/2 resulted in a remarkable increase in the number of cells that have visible neurites, and this effect was dose-dependent on AKTi-1/2 (Fig. 3B). This result together with that of the previous report implies that Akt can affect the ability of PC12 cells to neuronally differentiate in response to NGF.

We then measured the transcript levels of MafK, SytI, and Syn-1 using semi-quantitative PCR. The transcript level of each gene was proportionally increased by AKTi-1/2 (Fig. 3C). We therefore decided to use 10 μM AKTi-1/2 and analyzed the gene transcript levels using quantitative RT-PCR. AKTi-1/2 treatment increased the transcript levels of all three genes by 180-230% in PC12 (WT-Akt) cells (Fig. 3D) and by comparable amounts in PC12 (parental) cells (data not shown). These data, together, indicate that Akt downregulates the levels of *MafK*, *SytI*, and *Syn-1 *transcripts.

Since the overall level of a gene transcript is determined by transcription and mRNA stability, we investigated whether the elevated levels of gene transcripts in PC12 (DN-Akt) cells are due to increased mRNA stability in these Akt-downregulated cells. Gene transcript decay assays employing the transcriptional inhibitor actinomycin D revealed that the mRNA stability of MafK, *SytI*, and *Syn-1 *does not differ between PC12 (WT-Akt) and PC12 (DN-Akt) cells (data not shown). For this reason, the different levels of transcripts for these genes in PC12 (WT-Akt) and PC12 (DN-Akt) cells appear to reflect altered levels of transcription between the two cell populations.

### Effect of a Gsk3β inhibitor on expression of MafK, SytI, and Syn-1 genes in PC12 (DN-Akt) cells

**Figure 4:**
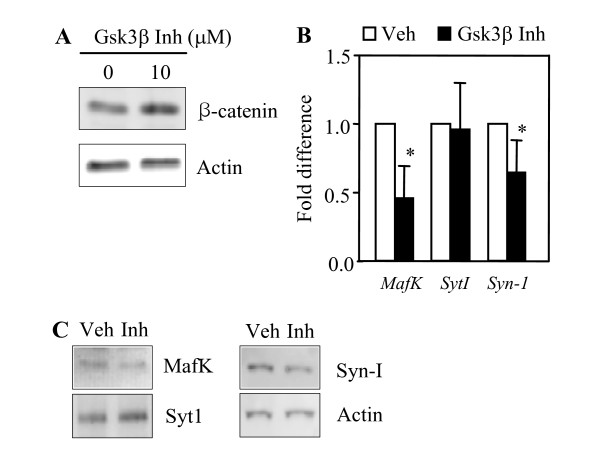
**Effect of a Gsk3β inhibitor on gene expression in PC12 (DN-Akt) cells**. PC12 (DN-Akt) cells were treated with 10 μM TWS119, a Gsk3β inhibitor for three hours. (A) Western blot analysis of β-catenin in PC12 (DN-Akt) cells treated with TWS119 to confirm that this agent works actively as a Gsk3β inhibitor in our experimental conditions. (B) mRNA levels for each gene are presented as a fold difference over that of vehicle-treated cells. *P < 0.05 (n = 3-4, mean + SD). (C) Western blot analysis of Syn-1 protein. Veh, vehicle; Inh, inhibitor.

Several transcription factors have been shown to be regulated by Akt and its downstream effectors. Gsk3β is one of the major molecules downstream of Akt. It phosphorylates and regulates transcription factors such as CREB and NF-kB [34]. We tested whether the Gsk3β pathway is involved in Akt-induced downregulation of transcription for the genes examined here. PC12 (DN-Akt) cells were treated with TWS119, a Gsk3β inhibitor [35]. Before examining the effect of this inhibitor on the transcript levels of the genes, we first wanted to determine whether this inhibitor would work actively as a Gsk3β inhibitor under our experimental conditions. To this end, we assayed the level of β-catenin, because β-catenin is another well-characterized substrate of Gsk3β and undergoes degradation upon phosphorylation [36]. As shown in Fig. 4A, TWS119 treatment of PC12 (DN-Akt) cells resulted in an increase in the level of β-catenin, indicating that TWS119 actively inhibits Gsk3β. Under these conditions, the transcript levels of *MafK *and *Syn-1 *were significantly lowered after treatment with this Gsk3β inhibitor, while that of *SytI *was not notably changed (Fig. 4B). This result suggests that the reduction of *MafK *and *Syn-1 *expression by Akt occurs, at least in part, through Gsk3β inhibition.

### Knockdown of Syn-1 decreases the neurite complexity in PC12 cells

**Figure 5:**
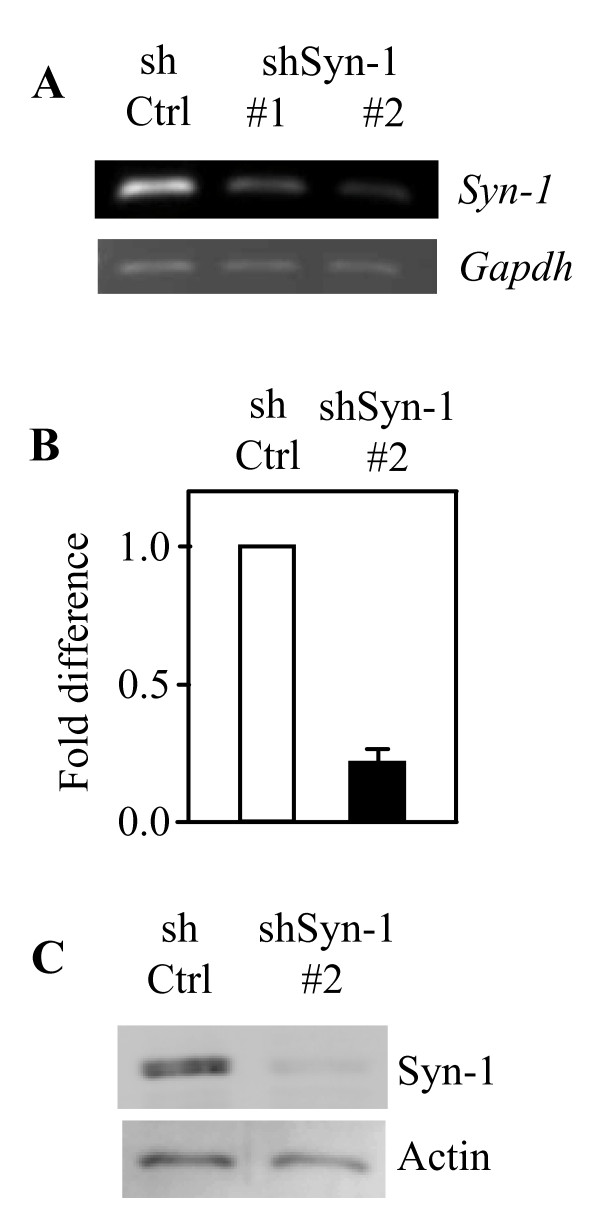
**shRNA expression vector against Syn-1 reduces the endogenous Syn-1 transcript and protein levels**. PC12 (DN-Akt) cells were infected with lentiviral shSyn-1#1, #2 or shControl (shCtrl). Syn-1 transcript was detected by semi-quantitative (A) and real-time (B) RT-PCR. (C) Western blot analysis.

**Figure 6:**
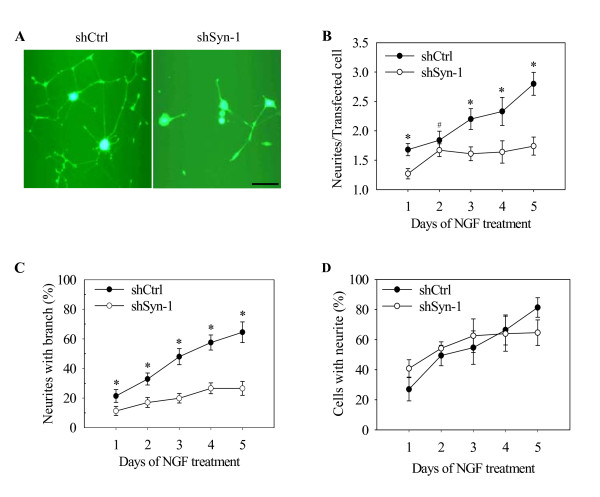
**Knockdown of endogenous Syn-1 by shRNA decreases the numbers of neurites and branches**. PC12 (DN-Akt) cells were infected with lentiviral shSyn-1#2 or shControl. (A) Fluorescent microscopic photographs of cells treated with NGF for 5 days. Scale = 50 μm. (B) The number of neurites originating from the neuritogenic cell body. (C) The percentage of branch-bearing neurites. (D) The percentage of neuritogenic cells among GFP-positive cells. Averages and standard deviations are derived from more than ten fields of view. * *P *< 0.001 and # *P *< 0.01 (n = 10-15, mean + SD).

We took an shRNA approach to knock down Syn-1 in PC12 (DN-Akt) cells. Cells were transfected with shControl, shSyn-1#1, or shSyn-1#2. By semiquantitative and quantitative RT-PCR and western blot analysis, we found an efficient knockdown of Syn-1 transcript and protein in cells transfected with shSyn-1#2 as compared with shControl-transfected cells (Fig. 5). We thus analyzed several aspects of neurite outgrowth in PC12 (DN-Akt) cells transfected with shSyn-1#2. It was microscopically observed that cells transfected with this shRNA possessed less elaborate neurites compared to control cells (Fig. 6A). The number of neurites originating from the cell body of neuritogenic cells was decreased in cells where Syn-1 was knocked down (Fig. 6B). Cells transfected with shSyn-1 had 1.74 ± 0.15 whereas control cells had 2.80 ± 0.20 neurites per cell on the 5^th ^day of NGF treatment. Another neurite outgrowth feature was also observed to be altered by Syn-1 knockdown. As for the neurite number, neurite branching was significantly reduced in cells transfected with shSyn-1 as compared with that of control cells (Fig. 6C). 64.5% of neurites from the control cells had at least one branch point, but only 26.5% of Syn-1-knockdown cell neurites displayed branching. Unlike these effects on the neurite network, however, the number of cells bearing at least one neurite was not altered noticeably by Syn-1 knockdown (Fig. 6D). These data demonstrate that Syn-1 does not affect neurite initiation but rather it promotes neurite profusion and branching in PC12 cells.

## Discussion

In this study, we identified novel Akt-regulated genes, *MafK*, *SytI*, and *Syn-1*. We showed that the transcript levels of these three genes were lower in PC12 (WT-Akt) cells than in PC12 (DN-Akt) cells, but increased following treatment with an Akt inhibitor. Taken together, these results indicate that Akt downregulates the expression of *MafK*, *SytI*, and *Syn-1*. MafK and SytI are involved in certain aspects of neuronal function. Inhibition of MafK expression has been reported to suppress neurite generation in PC12 cells treated with NGF and in primary immature neuronal cells [22], and mice deficient in MafK and MafG suffer from neurological dysfunction [37]. SytI is a synaptic vesicle protein in neurons. It acts as a calcium sensor and plays a role in neurotransmitter release [24,25]. Syn-1 interacts with a plethora of proteins via its PDZ domains and regulates transmembrane-receptor trafficking, tumor-cell metastasis, and probably neuronal-synaptic function [29]. The neuronal action of Syn-1 has been somewhat speculative and is based on the following findings. Syn-1 binds to several proteins implicated in axon outgrowth, fasciculation, and/or guidance, including Unc51.1 [38], ephrin receptors [39] and neurofascin [40]. Moreover, Syn-1 was shown to be expressed maximally in periods of intense growth and synapse formation during neuron development and its ectopic overexpression increased the number of short dendritic varicosities in neurons [30]. Here, we examined whether Syn-1 could affect the outgrowth of neurites. We transfected PC12 (DN-Akt) cells with a hairpin siRNA directed against Syn-1. This shRNA effectively reduced the endogenous level of Syn-1. We showed that a decreased Syn-1 level led to a decrease in the number of neurites and the percentage of branch-bearing neurites. Unlike these results, however, overexpression of Syn-1 in mature cultured hippocampal neurons was reported not to affect the number of branches [30]. This discrepancy may be due to the different levels of Syn-1 signaling, resulting from decreased or elevated expression of Syn-1, or reflect differences between mature hippocampal cells and neuroblast PC12 cells. When PC12 (DN-Akt) and PC12 (parental) cells were compared, it was also noticed that PC12 (parental) cells have fewer neurite branch points than PC12 (DN-Akt) cells (data not shown). These results suggest that Syn-1 has a role in producing profuse neurites. Though Syn-1 has been implicated in neuronal membrane varicosities [30], its Namikawa et al. 2000 K. Namikawa, M. Honma, K. Abe, M. Takeda, K. Mansur, T. Obata, A. Miwa, H. Okado and H. Kiyama, Akt/protein kinase B prevents injury-induced motoneuron death and accelerates axonal regeneration, *J. Neurosci*. **20 **(2000), pp. 2875-2886. View Record in Scopus | Cited By in Scopus (108)effect on neurite branching has not been described.

There have been conflicting reports regarding the role of Akt in neuronal outgrowth/differentiation. Several studies have reported a negative impact of Akt upon neuronal differentiation [2-6], while others published opposite results [41,42]. The reason for this discrepancy is not known. The direction of Akt's effect on neuronal differentiation probably depends on the state/context of cells. In fact, signaling molecules have been shown to induce different phenotypes, depending on the cellular stage/context. For instance, phosphoinositide 3-kinase has been shown to be required for NGF-induced differentiation of PC12 cells [43], however the same results were not observed by other research group [44]. It may be that the same signaling molecule has different sensitivity to its interacting partners under different cell contexts, producing different outcomes. It has been reported that Ras responds only to phosphoinositide 3-kinase of the basal rather than stimulated state under certain circiumstances [45]. Another example is Akt and Raf-MEK-MAPK pathways. Akt interacts with and inhibits the Raf-MEK-MAPK pathway [46], but this interaction occurs only in certain stages of cell differentiation [47]. The strength and duration of a molecule's signaling can be affected by its interacting proteins, which themselves also have changing activity and duration. Therefore, the signaling strength and duration of Raf-MAPK, which mediates neuronal differentiation when activated sustainedly [48,49], could be controlled by Akt. It has been suggested that a high level of Akt activity inhibits cell differentiation, whereas low Akt activation levels may be permissive or necessary for cell differentiation [2]. Furthermore, depending on the different level of Raf-MAPK, even the same level of Akt activity would produce different outcomes.

How Akt regulates neuronal outgrowth/differentiation has not been resolved. In the present study, we demonstrate that Akt downregulates the expression of several genes of neuronal functions, including *MafK*, *SytI*, and *Syn-1*, and some other genes such as *Canx *and *Cadm1 *(Table 2). It can be envisioned from these results that Akt-mediated downregulation of these genes of neuronal function is relevant to the diminished manifestation of neuronal phenotype in cells overexpressing Akt. Akt is often found activated in some neuroblastomas [50,51] as well as many other human cancers [52,53]. Neuroblastomas with hyperactive Akt or with low potential to differentiate in response to neurotrophins show poor prognosis [50,54]. There might be a causal connection between the Akt activity and the differentiation ability in these neuroblastomas. We observed that incubation of PC12 (WT-Akt) cells with the Akt inhibitor AKTi-1/2 restored their neurite outgrowth responsiveness to NGF (Fig. 3B). In this respect, a use of an Akt inhibitor like AKTi-1/2 would be advantageous in that it can not only possess the cytotoxic effect but can also lead the cells to respond to the differentiation effect of neurotrophins.

The mechanism of the downregulation of *MafK*, *SytI*, and *Syn-1 *genes by Akt remains unclear. However, the increased expression of *MafK *and *Syn-1 *genes in PC12 (WT-Akt) cells upon pharmacological inhibition of Gsk3β (Fig. 4) indicates that Gsk3β somehow regulates the transcription factors for these two genes. Gsk3β phosphorylates several transcription factors, including AP-1, CREB, HSF-1, NFAT, C/EBP, and NF-kB [34], Ngn2 [55], and Smad3/4 [56]. The functional consequence of this interaction with Gsk3β differs among transcription factors. While most are inhibited by Gsk3β, the transcriptional activity of C/EBP and Ngn2 is increased by Gsk3β. Since Akt phosphorylates and inhibits Gsk3β, the transcriptional activity of C/EBP and Ngn2 might be downregulated in PC12 (WT-Akt) cells but upregulated in PC12 (DN-Akt) cells. In this regard, it remains to be determined whether *MafK *and *Syn-1 *gene expressions are altered by expressing the active or dominant-negative (or silencing) form of C/EBP and Ngn2 in PC (WT-Akt) and PC (DN-Akt) cells, respectively. Our work indicates that the Gsk3β pathway does not appear to be implicated in the decreased expression of *SytI *in PC12 (WT-Akt). Expression of *SytI *in sympathetic neurons has been shown to be induced by BMP4 [27], and BMP4 signaling is known to be mediated by Smad proteins and MAPKs [57,58]. Therefore, it can be suggested that decreased expression of *SytI *in PC12 (WT-Akt) cells might be due to the Akt-mediated inhibition of the Raf-MAPK pathway. Alternatively, Akt might directly affect the transcription factor responsible for SytI expression. This could be Brn3, because this is the only transcription factor identified so far that could increase the expression of *SytI *in neuronal cells [59], and interestingly it has the preferred phosphorylation motif of Akt [31].

## Conclusion

Using PC12 cells expressing wild-type or dominant-negative Akt and also using a pharmacological inhibitor of Akt, we demonstrate that Akt can negatively affect the expression of *MafK*, *SytI*, and *Syn-1 *genes. MafK and SytI have been known to positively affect neuronal differentiation or neurotransmitter release. As for Syn-1, we observed here that Syn-1 has a role in producing profuse neurites. We also show that treatment with Akt inhibitor resulted in an improvement of neurite outgrowth. In summary, Akt regulates the expression of *MafK*, *SytI*, and *Syn-1*, which are all neuronal function-related genes.

## Competing interests

The authors declare that they have no competing interests.

## Authors' contributions

YR performed PCR experiments and drafted the manuscript. BJ performed all experiments. CYS helped BJ in experiments and participated in the writing of the manuscript. EUP aided in designing the experiments. CGK helped BJ in experiments. SY designed the study, coordinated the study, and participated in the writing of the manuscript.
